# Corticotomies as a surgical procedure to accelerate tooth movement during orthodontic treatment: A systematic review

**DOI:** 10.4317/medoral.21208

**Published:** 2016-07-31

**Authors:** Laura Fernández-Ferrer, José-María Montiel-Company, Eugenia Candel-Martí, José-Manuel Almerich-Silla, Miguel Peñarrocha-Diago, Carlos Bellot-Arcís

**Affiliations:** 1 Dentist, Department of Stomatology, Faculty of Medicine and Dentistry, University of Valencia, Spain; 2 Teaching Assistant, Department of Stomatology, Faculty of Medicine and Dentistry, University of Valencia, Spain; 3 Collaborating Lecturer, Master in Oral Surgery and Implant Dentistry, Department of Stomatology, Faculty of Medicine and Dentistry, University of Valencia, Spain; 4 Tenured Lecturer, Department of Stomatology, Faculty of Medicine and Dentistry, University of Valencia, Spain; 5 Head of Oral Surgery, Stomatology Department, Faculty of Medicine and Dentistry, University of Valencia, Spain; 6 Associate Lecturer, Department of Stomatology, Faculty of Medicine and Dentistry, University of Valencia, Spain

## Abstract

**Background:**

One of the main aims of orthodontists is to reduce the treatment time as much as possible, particularly in view of the rise in demand for orthodontic treatment among adult patients. The objective of this systematic review was to examine the effectiveness of corticotomy as a surgical procedure that accelerates orthodontic tooth movement, together with its possible adverse effects.

**Material and Methods:**

A systematic review of articles in 4 databases, Pubmed, Cochrane, Scopus and Embase, complemented by a manual search, identified 772 articles. The duplicates were eliminated and a critical reading of titles and abstracts led to the rejection of articles that did not meet the objectives of the review, leaving 69. After reading the full text of these articles, 49 were excluded because they did not meet the inclusion criteria. On applying the CONSORT criteria as a quality filter, a further 4 were eliminated due to low quality. Finally, 16 articles (4 systematic reviews and 12 controlled trials) were reviewed.

**Results:**

All the studies agree that corticotomy prior to orthodontic treatment accelerates dental movement, reducing the treatment time. With regard to side-effects, no periodontal damage was found, although this was only studied in the short term.

**Conclusions:**

The evidence regarding the results of corticotomy is limited, given the small number of quality clinical studies available. Before this procedure is included as a routine practice in dental surgeries, studies of higher methodological quality are required, studying a greater number of individuals and examining the possible long-term adverse effects and the cost/benefit of the procedure.

**Key words:**Corticotomy, orthodontics, adults, accelerated tooth movement, osteotomy.

## Introduction

The length of treatment is one of the patients’ main concerns, particularly among adults. Consequently, one of the main aims of orthodontists is to reduce the treatment as much as possible (1,2). However, this is not just an aesthetic and functional demand, as reducing the treatment time is also necessary to avoid the incidence of adverse effects such as root resorption, oral hygiene difficulties or the appearance of caries (2,3).

The mean duration of orthodontic treatment is between 16 and 18 months, but depends on many factors, such as the malocclusion itself, whether or not extractions are required, gender, age, cooperation from the patient and clinical experience (2,4). In cases involving maximum anchorage need owing to extraction of the maxillary premolars, the total treatment time rises to 18-24 months (5). When the orthodontic treatment is carried out in preparation for orthognathic surgery, the treatment time can increase to 3 years (4,6,7).

As a result, researchers have investigated new techniques that are combined with orthodontic treatment to accelerate tooth movement. These techniques can be divided into surgical and non-surgical procedures. The latter include low-intensity laser therapy, photobiomodulation, pulsed electromagnetic fields, electric currents and pharmacological intervention (2,7-9). The surgical methods include corticotomies, with different designs and modifications, and periodontal ligament distraction (10).

Corticotomy is defined as the surgical procedure that intentionally inflicts mechanical damage on the cortical bone. This increases bone remodelling to accelerate the repair and achieve functional recovery. The process takes place through recruiting osteoblasts and osteoclasts activated by local intercellular mediators (6,8,11). This creates a transitory state of osteoporosis, characterised by a reduction in bone density, which causes less resistance to tooth movement (6,12). This phenomenon was described by Harold Frost, who named it the Regional Acceleratory Phenomenon (RAP) (11).

The corticotomy technique dates back to 1983 and has been revised and modified over the years to eliminate the possible risks of the procedure (3). Köle (13) described a procedure named Bone Block Movement, in which cuts were made around the tooth in such a way that it remain anchored in a block of bone, which was what moved (1,6). In 1998, Liou & Huang (14) introduced the periodontal ligament distraction technique, which remodels the socket after extraction and uses a custom-made distraction device to effect canine retraction (15). Wilcko *et al.* coined the term ‘accelerated osteogenic orthodontics’, also known as Wilckodontics. (16) This modification of existing corticotomies involved full flap lifting both labially and lingually and selective decortication followed by bone grafting. Wilcko *et al.* postulated the creation of a time window of a few months in which the teeth can be moved more rapidly (16).

However, the evidence is still inconclusive. Owing to the importance of this subject for both clinicians and patients and the continuous evolution of the techniques employed, it was decided to conduct an up-to-date systematic review to examine the effect of corticotomies on tooth movement during orthodontic treatment and their possible adverse effects on the tooth, the periodontal tissues and/or the patient.

## Material and Methods

The bibliography was reviewed systematically, following the PRISMA (Preferred Reporting Items for Systematic Reviews and Meta-Analyses) recommendations (17).

- Eligibility criteria

The selection criteria for inclusion were: articles, articles in press and reviews concerning studies in humans. Only systematic reviews, meta-analyses and randomised controlled trials (RCTs) were accepted. All the studies included compared an experimental group with a control group. Those employing non-surgical methods were discarded. The control group consisted of a comparable group of patients who only received conventional orthodontic treatments or who received a modification of the treatment method used for the experimental group. The inclusion criteria were studies conducted in humans who required orthodontic treatment, with no age limit. Articles that included patients with a syndrome or systemic illness or who were taking medicines that could affect orthodontic movement were excluded. The primary results collected were all those that referred to the speed of tooth movement or the length of orthodontic treatment time. The secondary results were the possible adverse effects of the surgical procedure.

- Information sources and search strategy 

To identify the most relevant studies irrespective of language, a rigorous electronic search was made in the Pubmed, Scopus, Cochrane Library and Embase databases and was updated in June 2015. The search strategy was based on the combination of 14 primary terms concerning the surgical technique employed (“Corticotomy” or “corticotomy-assisted” or “corticotomy-facilitated”, “Piezocision”, “Micro-osteoperforations” or “MOPS”, “Piezosurgery”, “Osteotomy” or “osteotomy-assisted” or “osteotomy-facilitated”, “Osteogenic orthodontics”, “Periodontally accelerated osteogenic orthodontics” or “PAOO”, and “Dentoalveolar distraction”) and 7 secondary terms regarding tooth movement (“Speedy orthodontics”, “Orthodontics”, “Dental movement”, “Accelerated tooth movement”, “Rapid tooth movement”, “Regional accelerated phenomenon” or “RAP”). All the possible combinations between the two columns were explored and the references cited in the articles included were then searched for studies that had not been found during the primary search.

- Study selection, data extraction and list of variables

Two independent reviewers assessed the titles and abstracts of all the articles selected. If they disagreed, a third reviewer was consulted. When the information in the abstract was insufficient to reach a conclusion, the reviewers read the full article before taking the final decision. The reviewers then read the full text of all the articles.

The following variables were extracted from each article: author and year of publication, type of study, sample characteristics, objectives, follow-up time, results, and characteristics of the procedures followed.

- Study quality

The trials were classified by quality (high, medium or low) according to the CONSORT criteria (18) adapted by Mattos *et al.* (19), as used by a number of authors of other systematic reviews for the same purpose (20-22). This is a reduced list of 9 criteria, out of the original 27, that assess the most important points for classifying the quality of the methods, design, execution and analysis of each study. Those of low quality were excluded. The quality of the systematic reviews was assessed through a synthesis of the PRISMA guidelines (17).

- Measurement of the variables and synthesis of the results

The means and confidence intervals of the variables analysed were taken into account wherever possible. Combined assessment of the results was intended if the homogeneity of the studies made this possible.

## Results

1. Study selection and flow diagram

**Figure 1:**
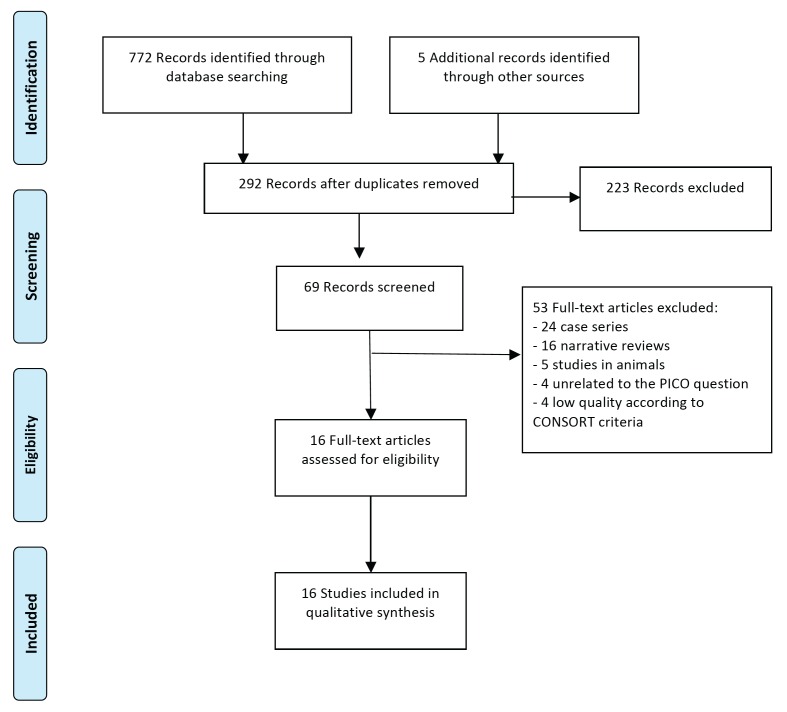
Flow Diagram.

Following a thorough search, 96 articles were identified in Medline, 469 in Scopus, 11 in the Cochrane Library and 196 in Embase, totalling 772 articles. After eliminating the 485 duplicates, a further 287 were excluded on reading the title and abstract (163 were unrelated to the research question and 60 did not meet the inclusion criteria). The remaining 64 articles were read and analysed. A further 5 references that had not been found through the primary search were added manually. On reading these 69 articles, 53 were excluded for the following reasons: 24 were case series, 16 were narrative reviews, 5 were studies in animals, 4 were unrelated to the research question and 4 were considered of low quality according to the CONSORT criteria. The 16 articles that met the inclusion criteria and were of medium to high quality were included (Fig. 1).

2. Characteristics of the studies

Of the 16 articles included, 4 were systematic reviews and 12 were studies. Of the studies, 5 were of high quality and 7 of medium quality. The 4 reviews all met the PRISMA criteria ( Table 1).

**Table 1:**
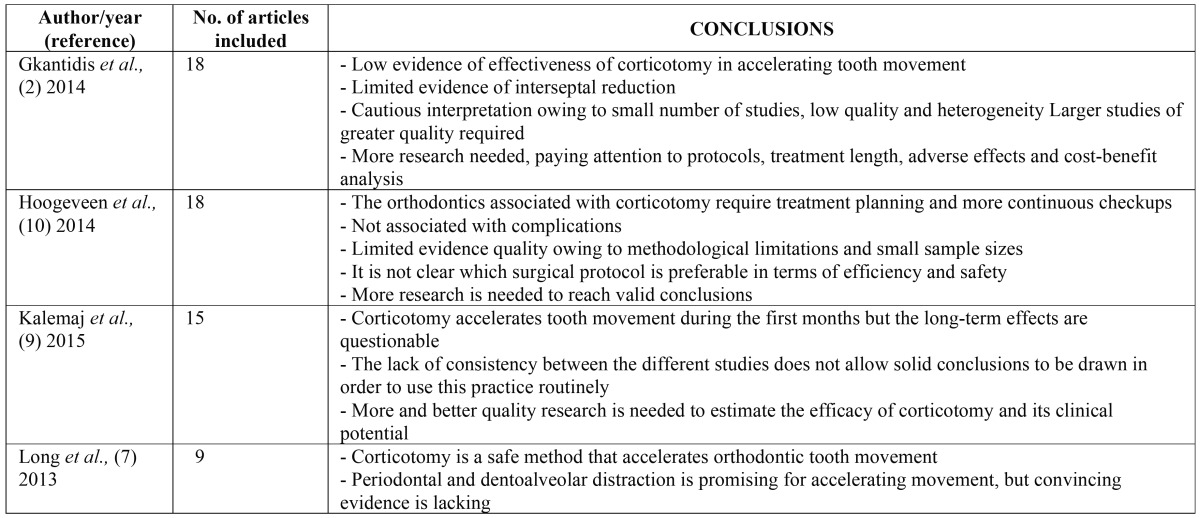
Conclusions of the systematic reviews included.

Nine of the 12 studies compared an experimental group, treated with orthodontics and corticotomy, with a control group. The primary objectives of 5 of the trials were to study the speed of tooth movement (1,5,6,23,24). A further 5 measured the orthodontic treatment time (4,8,24-26). The secondary objectives were to examine the effect of corticotomies on periodontal tissues (3,5,6,8,24,26), postoperative pain (1,15,23,25), loss of posterior anchorage (4,6), inflammation markers (23), root length (3,26) and canine rotation and tipping (5) ( Table 2 and  Table 2 continue ).

**Table 2:**
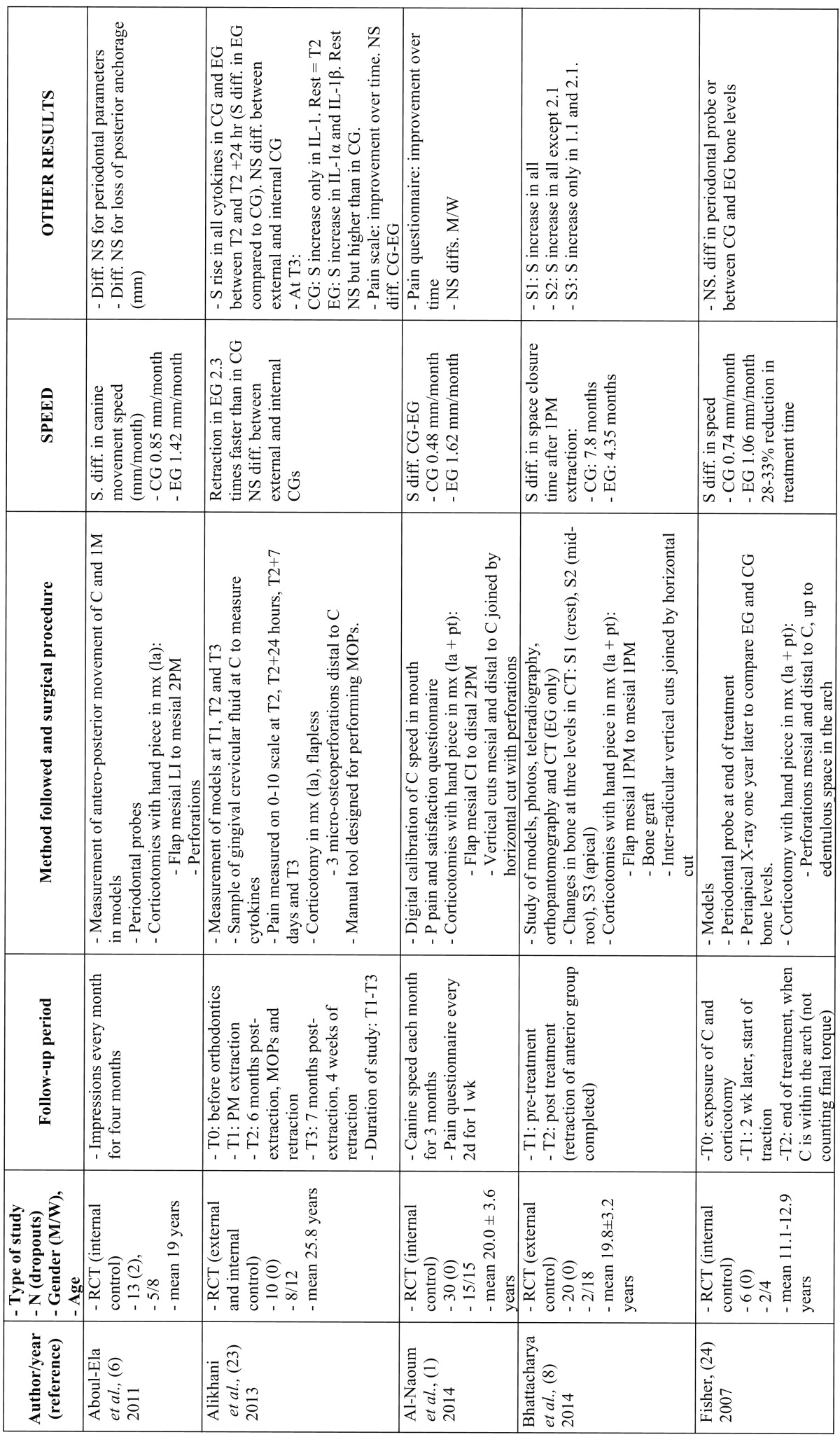
Studies that assess the speed of tooth movement and the duration of orthodontic treatment.

**Table 2 continue:**
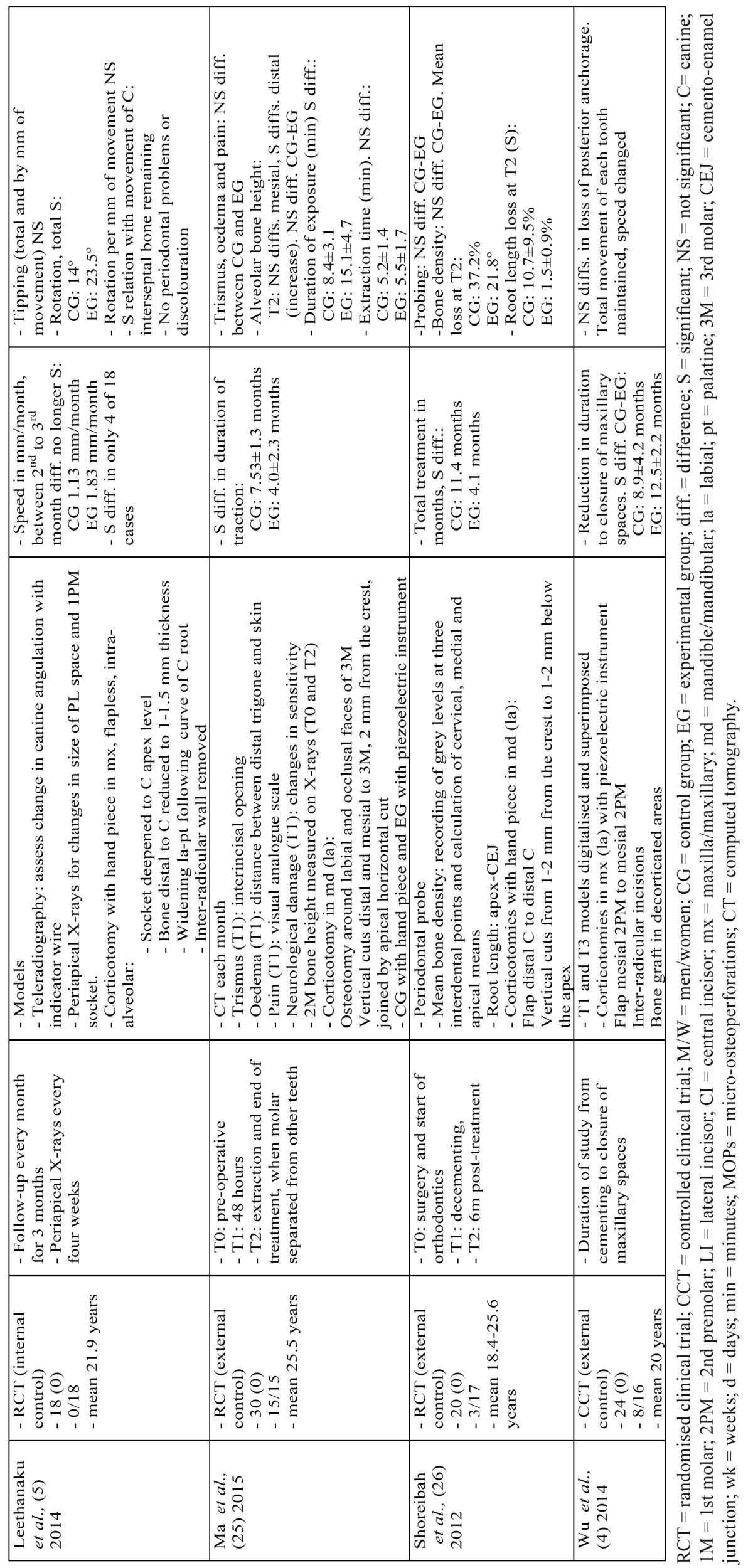
Studies that assess the speed of tooth movement and the duration of orthodontic treatment.

In the other 3 studies, corticotomy was employed in both groups. Cassetta *et al.* (27) used the OHIP-14 questionnaire to examine the impact of the corticotomy on the patients’ quality of life, Shoreibah *et al.* (3) studied the effect of bone grafting on the decorticated areas and Mowafy *et al.* (15), the only researchers to use a dentoalveolar distraction device to retract the anterior group, assessed the movement and tipping of the canines and the perception of pain ( Table 3).

**Table 3:**
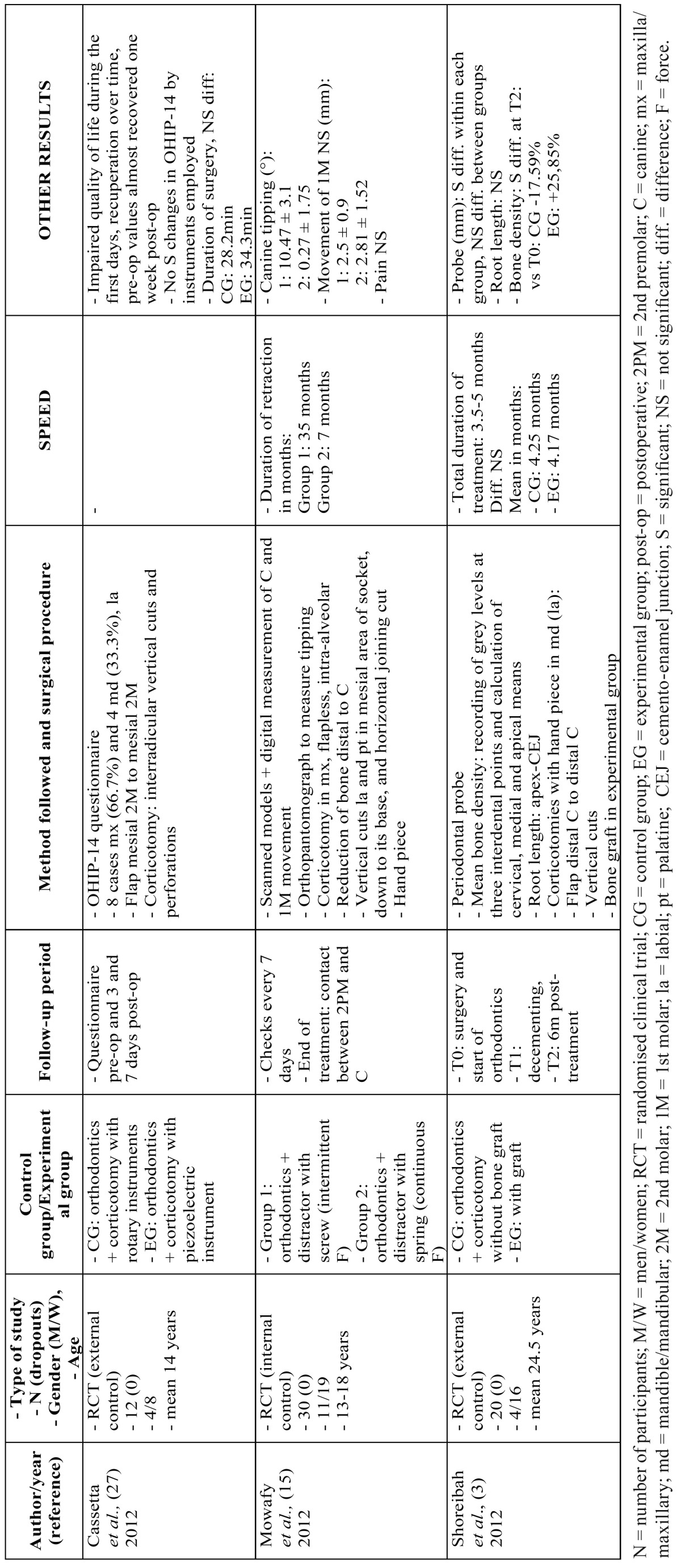
Studies that assess other aspects of corticotomy: quality of life, periodontal distractors and effects of bone grafting.

 Table 2 and  Table 2 continue continue and  Table 3 show the data from all the studies, including the type of surgical procedure, its design and the instruments used, and the chronological order of the procedures. They also show the general characteristics of the sample, the results, the follow-up time and the methods used to measure the variables.

3. Qualitative and quantitative synthesis of the studies included

Of the 5 trials that studied the speed of tooth movement, 4 extracted the first maxillary premolars and retracted the anterior group. Fisher (24) is the only study that cannot be compared with the others, as its objective and methodology were different. Among the other 4 studies, 3 were of high quality (1,5,23) and 1 of medium quality (6). In all of these, corticotomy was performed in one quadrant and the other quadrant acted as the control. Concerning the design of the surgery, 2 perforated the cortical bone labially, one raising a flap from the lateral incisor to the second premolar (6) and the other only performing three micro-osteoperforations through the gum (23). In the study by Al-Naoum *et al.* (1), flaps were raised both labially and palatally and as well as the perforations, vertical cuts were made mesially and distally to the canine and were joined by a horizontal cut at apical level. Leethanakul *et al.* (5) did not raise flaps but performed a surgical modification in the socket of the extracted premolar. Retraction began in all the patients on the same day as surgery, but the force applied varied. The comparable results of all the studies are those of the first month after commencing retraction. All the studies concluded that the canine moved more rapidly in the corticotomy group. The mean was 2.6 times faster. The increase in the speed of movement ranged from 1.8 (5) to 3.8 times faster (1).

The studies by Wu *et al.* (4) and Bhattacharya *et al.* (8) only measured the length of time taken to close the extraction spaces. The treatment time was faster in the corticotomy group. Shoreibah *et al.* (26) studied the total treatment duration, which was almost 3 times shorter in the experimental group. Fisher (24) also measured total duration, which was reduced by between 28% and 33%. In the study by Ma *et al.* (25), the time was reduced by nearly 50%.

The secondary effects of corticotomy have only been studied in the short term and none of the studies have encountered periodontal damage (3.5,^6^,^24^,^26^).

Regarding changes to the bone, inserting bone grafts in the decorticated areas does increase the bone density following treatment to a significant degree (3,8). In studies where no grafting took place, Shoreibah *et al.* (26) found a generalised loss of bone density in both the corticotomy group and in the control group, with no significant differences between them.

Pain is another adverse effect investigated by several studies, but none found any differences between the corticotomy patients and the control group (1,15,23,25,27). All reported increased pain initially followed by improvement over time.

Loss of anchorage was only studied by Wu *et al.* (4) and Aboul-Ela *et al.* (6) in plaster models. Both concluded that the loss of anchorage did not depend on the corticotomy. Differences in canine tipping were not statistically significant according to Leethanakul *et al.* (5). As regards loss of root length, Shoreibah *et al.* (3,26) concluded that this was greater when corticotomy was not performed.

Only the study by Alikhani *et al.* (23) studied the effect of micro-osteoperforations on certain inflammation markers, all related with the differentiation of osteoclast precursor cells. A significant increase occurred 24 hours after the start of treatment in both the control and the experimental group, but was significantly higher in the latter. One month later, the levels remained higher in the experimental group.

## Discussion

As the length of the orthodontic treatment is one of the patients’ main concerns, the interest in methods to accelerate tooth movement has increased. To assess the increased speed of movement following corticotomy, the different studies need to be examined. Of the 12 trials with a medium to high methodological quality included in the present review, only 4 employed comparable methods. However, all of them found that movement is always faster following corticotomy. The theory that enjoys the widest acceptance is Frost’s RAP (Regional Acceleratory Phenomenon). The study by Alikhani *et al.* (23) revealed that micro-osteoperforations increase the expression of cytokines and chemotactins. These amplify the inflammatory response, causing greater bone turnover and increasing the speed of movement, which supports Frost’s theory. However, histological studies are needed to confirm this period of transitory osteoporosis (1,6).

The study by Al-Naoum *et al.* (1) found the greatest speed of movement in the canines of the experimental group (between 2 and 4 times faster). This study and that of Bhattacharya *et al.* (8) were those with the most invasive design. Frost (11) considered that the greatest resistance to tooth movement was due to the cortical walls. Consequently, breaking them up would reduce the treatment time. Fisher (24) also considered that corticotomy encourages faster tooth movement because it reduces the bone mass. It would therefore be logical to expect that the more corticotomy performed, the better the result. The results of Bhattacharya *et al.* (8) agreed with those of other articles in which the tooth in question moved twice as fast when corticotomy had been performed. Even studies that did not involve flaps, such as that of Alikhani *et al.* (23), obtained similar figures. It would therefore appear that the increase in speed is not influenced by the extent of the corticotomy. This would also benefit the patient, due to a shorter operating time and a better post-operative period (6).

The only studies that found a significant increase in post-operative pain following corticotomy were those by Al-Naoum *et al.* (1) and Cassetta *et al.* (27), who had used more invasive surgical procedures. The most promising studies at present are those which did not involve flaps, as the procedure is simpler and apparently just as effective and the post-operative conditions are better (5).

Leethanakul *et al.* and Mowafy *et al.* (5,15) carried out intra-alveolar surgical modification after extracting the first premolars, reducing the resistance of the bony interseptal wall in order to facilitate the movement of the canine. However, the evidence for this intervention is scanty. Leethanakul *et al.* (5) concluded that the only significant factor correlated with canine movement is the quantity of bone remaining in the mesial wall of the socket. However, only 4 out of the 18 patients exhibited clinically significant results, with spaces closing in three months. As a result, these authors concluded that there are other factors that influence movement, such as the root anatomy of the canine. Consideration of these factors means that the technique is sensitive to case selection and does not always achieve the expected result (5).

Mowafy *et al.* (15) and Alikhani *et al.* (23) studied tipping after corticotomy and concluded that there were no significant differences between the experimental and control groups. This could constitute a serious limitation for the studies reviewed, as if they did not take tipping into account they may have underestimated the treatment time by not counting the extra time required to correct the angulation.

The orthodontic force applied is another of the variables that make it difficult to compare the results of all the studies. Nevertheless, there were no differences between the results of Alikhani *et al.* (23) and those of Bhattacharya *et al.* (8), who used 2.5 times more force.

In order to establish an appropriate surgical protocol, the extent of the corticotomy required also needs to be assessed.

The chronological order of the treatment stages and the moment when surgery and orthodontics are initiated are also decisive. In almost all the studies, orthodontics were initiated on the same day as surgery, but three began the orthodontic treatment two weeks afterwards (4,8,24). This could be one of the reasons why Bhattacharya *et al.* (8) did not find a greater difference between the experimental and control groups. Aboul-Ela *et al.* (6), Al-Naoum *et al.* (1) and Leethanakul *et al.* (5) reported that the speed increased in the first two months but fell as time passed, drawing level with that of the control group. One possible explanation is that three months after the extraction the tooth socket fills up with regenerated bone tissue and opposes greater resistance to movement in spite of the RAP (5). Alikhani *et al.* (23) concluded that the extractions also raise the level of inflammation markers and should therefore be delayed as much as possible until the moment of greatest tooth movement is reached.

Although all the studies included were of medium or high quality according to the CONSORT criteria (20), the sample sizes were small in all cases, so the evidence they provide is not very strong. Also, the follow-up periods were short, so did not show any adverse effects in the medium and long-term (5,23). Future studies need to increase the sample size (4,8) and establish surgical and orthodontic protocols. The protocol needs to establish the following parameters: appropriate case selection, chronological order of treatment, surgery designed to achieve maximum efficacy with the minimum extent of intervention, with or without bone grafting depending on the needs of each patient, using the optimum orthodontic forces in relation to the working area, checkups at shorter intervals, whether or not temporary anchorage is needed, and X-rays on finishing the treatment to check whether mass movement has taken place.

With the information available to date, corticotomy cannot be recommended systematically even though it offers advantages related to the shorter duration of orthodontic treatment, such as a lower incidence of apical resorption. However, an evidence-based protocol is still not available to ensure the most effective treatment possible.

At present, clinicians should limit their use of this technique to specific cases such as when a patient already needs surgery (periodontal surgery or traction of impacted canines or third molars) or will be undergoing orthognathic surgery, when reducing the treatment time is of the utmost importance. The most promising surgical treatments are the least invasive ones, such as micro-osteoperforations (22), and further research should therefore follow this line of investigation.

## Conclusions

Within the limitations of this review, the results of the studies included confirm that combining conventional orthodontic treatment with corticotomy reduces the duration of the treatment by accelerating tooth movement. However, few clinical trials have been conducted to date in this area, with small samples of patients and short-term follow-up, so the efficiency-safety ratio is not conclusive.

Before this procedure is included as a routine practice in dental surgeries, studies of higher methodological quality are required, studying a greater number of individuals and examining the possible long-term adverse effects and the cost/benefit ratio of the procedure.

## References

[1] Al-Naoum F, Hajeer MY, Al-Jundi A (2014). Does alveolar corticotomy accelerate orthodontic tooth movement when retracting upper canines? A split-mouth design randomized controlled trial. J Oral Maxillofac Surg.

[2] Gkantidis N, Mistakidis I, Kouskoura T, Pandis N (2014). Effectiveness of non-conventional methods for accelerated orthodontic tooth movement: A systematic review and meta-analysis. J Dent.

[3] Shoreibah EA, Samir A, Ibrahim A, Attia MS, Diab MMN (2012). Clinical and radiographic evaluation of bone grafting in corticotomy-facilitated orthodontic in adults. J Int Acad Periodontol.

[4] Wu JQ, Jiang JH, Xu L, Liang C, Bai YY, Zou W (2015). A pilot clinical study of class III surgical patients facilitated by improved accelerated osteogenic orthodontic treatments. Angle Orthod.

[5] Leethanakul C, Kanokkulchai S, Pongpanich S, Leepong N, Charoemratrote C (2014). Interseptal bone reduction on the rate of maxillary canine retraction. Angle Orthod.

[6] Aboul-Ela SM, El-Beialy AR, El-Sayed KM, Selim EM, El-Mangoury NH, Mostafa YA (2011). Miniscrew implant-supported maxillary canine retraction with and without corticotomy-facilitated orthodontics. Am J of Orthod Dentofacial Orthop.

[7] Long H, Pyakurel U, Wang Y, Liao L, Zhou Y, Lai W (2013). Interventions for accelerating orthodontic tooth movement. A systematic review. Angle Orthod.

[8] Bhattacharya P, Bhattacharya H, Anjum A, Bhandari R, Agarwal DK, Gupta A (2014). Assessment of Corticotomy Facilitated Tooth Movement and Changes in Alveolar Bone Thickness - A CT Scan Study. J Clin Diagn Res.

[9] Kalemaj Z, Debernardl CL, Buti J (2015). Efficacy of surgical and non-surgical interventions on accelerating orthodontic tooth movement: A systematic review. Eur J Oral Implantol.

[10] Hoogeveen EJ, Jansma J, Ren Y (2014). Surgically facilitated orthodontic treatment: A systematic review. Am J Orthod Dentofacial Orthop.

[11] Frost HM (1983). The regional acceleratory phenomenon: a review. Henry Ford Hosp Med J.

[12] Hassan AH, Ahmad A, Fraidi A, Samar H (2010). Corticotomy-assisted orthodontic treatment: Review. Open Dent J.

[13] Kole H (1959). Surgical operations on the alveolar ridge to correct occlusal abnormalities. Oral Surg Oral Med Oral Pathol.

[14] Liou EJ, Huang CS (1998). Rapid canine retraction through distraction of the periodontal ligament. Am J Orthod Dentofacial Orthop.

[15] Mowafy MI, Zaher AR (2012). Anchorage loss during canine retraction using intermittent versus continuous force distractions; a split mouth randomized clinical trial. Prog Orthod.

[16] Wilcko W, Wilcko MT (2013). Accelerating tooth movement: The case for corticotomy-induced orthodontics. Am J of Orthod Dentofacial Orthop.

[17] Liberati A, Altman DG, Tetzlaff J, Mulrow C, Gotzsche PC, Ioannidis JP (2009). The PRISMA statement for reporting systematic reviews and meta-analyses of studies that evaluate healthcare interventions: explanation and elaboration. BMJ.

[18] Schulz KF, Altman DG, Moher D, Consort Group (2010). Consort 2010 statement: updated guidelines for reporting parallel group randomised trials. BMJ.

[19] Mattos CT, Vilani GNL, Sant'Anna EF, Ruellas ACO, Maia LC (2011). Effects of orthognathic surgery on oropharyngeal airway: a meta-analysis. Int J Oral Maxillofac Surg.

[20] Fernández-Ferrer L, Montiel-Company JM, Pinho T, Almerich-Silla JM, Bellot-Arcís C (2015). Effects of mandibular setback surgery on upper airway dimensions and their influence on obstructive sleep apnoea - a systematic review. J Craniomaxillofac Surg.

[21] Serra-Torres S, Bellot-Arcís C, Montiel-Company JM, Marco-Algarra J, Almerich-Silla JM (2016). Effectiveness of mandibular advancement appliances in treating obstructive sleep apnea syndrome: A systematic review. Laryngoscope.

[22] Bermell-Baviera A, Bellot-Arcís C, Montiel-Company JM, Almerich-Silla JM (2016). Effects of mandibular advancement surgery on the temporomandibular joint and muscular and articular adaptive changes--a systematic review. Int J Oral Maxillofac Surg.

[23] Alikhani M, Raptis M, Zoldan B, Sangsuwon C, Lee YB, Alyami B (2013). Effect of micro-osteoperforations on the rate of toot movement. Am J of Orthod Dentofacial Orthop.

[24] Fischer TJ (2007). Orthodontic treatment acceleration with corticotomy-assisted exposure of palatally impacted canines. Angle Orthod.

[25] Ma Z, Xu G, Yang C, Xie Q, Shen Y, Zhang S (2015). Efficacy of the technique of piezoelectric corticotomy for orthodontic traction of impacted mandibular third molars. Br J Oral Maxillofac Surg.

[26] Shoreibah EA, Salama AE, Attia MS, Abu-Seida SM (2012). Corticotomy-facilitated orthodontics in adults using a further modified technique. J Int Acad Periodontol.

[27] Cassetta M, Di Carlo S, Giansanti M, Pompa V, Pompa G, Barbato E (2012). The impact of osteotomy technique for corticotomy-assisted orthodontic treatment (CAOT) on oral health-related quality of life. Eur Rev for Med Pharmacol Sci.

